# Transgenic Cavendish bananas with resistance to Fusarium wilt tropical race 4

**DOI:** 10.1038/s41467-017-01670-6

**Published:** 2017-11-14

**Authors:** James Dale, Anthony James, Jean-Yves Paul, Harjeet Khanna, Mark Smith, Santy Peraza-Echeverria, Fernando Garcia-Bastidas, Gert Kema, Peter Waterhouse, Kerrie Mengersen, Robert Harding

**Affiliations:** 1 Centre for Tropical Crops and Biocommodities, Queensland University of Technology, Brisbane, 4001 Queensland Australia; 2 Darwin Banana Farming Company, Lambells Lagoon, 0822 Northern Territory Australia; 3 Wageningen University and Research Centre, Plant Research International, Wageningen, 6700 The Netherlands; 4 Science and Engineering Faculty, Queensland University of Technology, Brisbane, 4001 Queensland Australia; 5 Present Address: Sugar Research Australia, Indooroopilly, 4068 Queensland Australia; 6 Present Address: Unidad de Biotecnologia Centro de Investigacion Cientifica de Yucatan, Merida, 97205 Yucatan Mexico

## Abstract

Banana (*Musa* spp.) is a staple food for more than 400 million people. Over 40% of world production and virtually all the export trade is based on Cavendish banana. However, Cavendish banana is under threat from a virulent fungus, *Fusarium oxysporum* f. sp. *cubense* tropical race 4 (TR4) for which no acceptable resistant replacement has been identified. Here we report the identification of transgenic Cavendish with resistance to TR4. In our 3-year field trial, two lines of transgenic Cavendish, one transformed with *RGA2*, a gene isolated from a TR4-resistant diploid banana, and the other with a nematode-derived gene, *Ced9*, remain disease free. Transgene expression in the RGA2 lines is strongly correlated with resistance. Endogenous RGA2 homologs are also present in Cavendish but are expressed tenfold lower than that in our most resistant transgenic line. The expression of these homologs can potentially be elevated through gene editing, to provide non-transgenic resistance.

## Abstract (web summary)

The newly recognized Fusarium wilt pathogen tropical race 4 is threatening worldwide banana production. Here, the authors transform Cavendish bananas with a resistance gene, *RGA2*, from diploid banana or a nematode-derived gene, *Ced9*, and confer resistance to natural infection under field conditions.

## Introduction

Fusarium wilt or Panama disease is a devastating disease of bananas. In the first half of last century, it caused one of the most serious plant disease epidemics in history. During that period, *F. oxysporum* f. sp. *cubense* (Foc), the fungus responsible for Fusarium wilt, caused a major epidemic in commercial banana plantations in South and Central America in the then dominant export cultivar Gros Michel^1^. This epidemic was caused by Foc race 1 and led to the almost complete replacement of Gros Michel with Cavendish, which is resistant to Foc race 1. Cavendish now accounts for >40% of world's banana production and completely dominates the banana export market, which amounts to 15% of world production. Despite this, Foc race 1 continues to cause significant disease in a wide range of other locally produced and traded banana cultivars^2^. Foc invades through the roots before entering the corm and pseudostem where it causes extensive necrosis leading to plant death^3^. The fungus is disseminated in infested soil, infected planting material and water including irrigation water and floods, and can remain in the soil for >40 years^1^. In the early 1990s, another form of Foc, tropical race 4 (TR4) was recognized in South East Asia^4^, which differed from Foc race 1 in that it infects and kills Cavendish as well as a number of other important race 1-resistant cultivars. Foc TR4 now devastates Cavendish plantations in Indonesia, Malaysia, China, the Philippines, Australia, and Mozambique. It continues to move internationally with recent reports of its spread into Jordan, Pakistan, and Lebanon^4,5^, and it is highly likely that the fungus and the disease will continue to spread particularly in south and south east Asia. Of the banana-producing continents, only the Americas have yet to record TR4. The disease now poses a very significant threat to commercial banana production worldwide and, together with race 1, severely limits the number of banana cultivars suitable for either large scale or smallholder production.

There is no effective chemical control for TR4^4^ and efforts to contain the disease through inter- or intra-national quarantine have clearly been ineffective as evidenced by the continued spread between continents, countries, and regions. Locally, efforts such as destruction of infected plants, isolation of infested areas, and disinfection of vehicles, machinery, and clothing are likely, at best, to slow the rate of spread. Although somaclonal variants of Giant Cavendish (Giant Cavendish Tissue Culture Variants (GCTCVs)) with varying levels of tolerance to TR4 have been generated in Taiwan through tissue culturing^6^, these are considered a short-term solution to disease control at best due to lack of immunity and undesirable agronomic traits^7^. The lack of effective TR4 control measures and the devastating impact of the disease make the deployment of resistance genes an obvious and attractive strategy. Here we report the generation and field-trialling of transgenic Cavendish banana plants and the identification of lines with robust resistance to TR4.

## Results

### Generation and characterization of transgenic banana plants

**Fig. 1:**
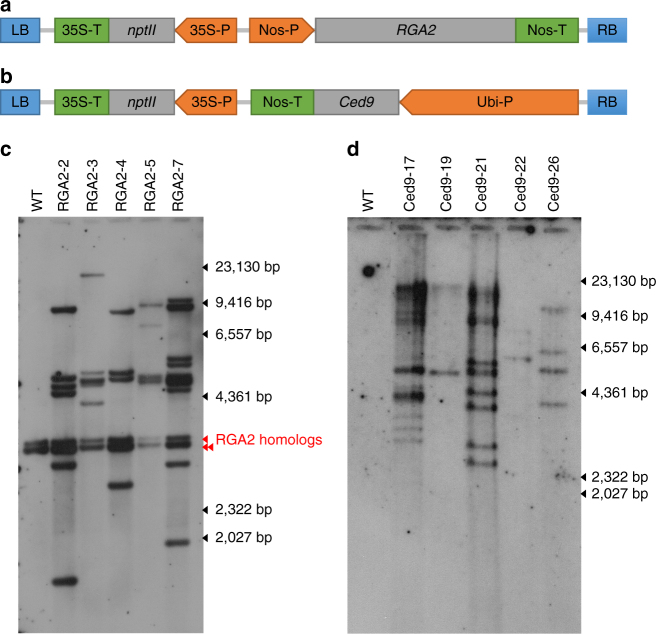
Transgene expression cassettes and Southern analysis of selected transgenic lines. **a** RGA2 and **b** Ced9 expression cassettes. LB, left border; RB, right border. Determination of transgene copy number in **c** *RGA2* and **d** *Ced9* transgenic banana lines by Southern blot analysis. Genomic DNA from WT, *RGA2* and *Ced9* lines was digested with *Hin*dIII and *Xma*I, respectively. DNA molecular weight marker II (Roche) reference is indicated on the right hand side

We have previously shown that the *Ced9* anti-apoptosis gene derived from the nematode *Caenorhabditis elegans* can confer resistance to Foc race 1 in transgenic Lady finger bananas^8^. We have also previously isolated resistance gene analog 2 (*RGA2*), a putative nucleotide-binding and leucine-rich repeat (NB-LRR)-type resistance (R) gene, from a seedling of *Musa acuminata* ssp. *malaccensis* with resistance to TR4^9^. Sequence analysis of this gene^7^ revealed a close phylogenetic relationship to the NB-LRR type genes, including *I2* ^10^ and *Fom-2* ^11^, which have been shown to encode Fusarium wilt resistance in tomato and melon, respectively. Therefore, we constructed both *Ced9-* and *RGA2*-derived transgene expression cassettes (Fig. 1a,b) where *Ced9* was under the control of the maize polyubiquitin promoter (Ubi-P) and *RGA2* was under the control of the Agrobacterium *nopaline synthase* promoter (Nos-P). These cassettes were transformed separately into the Cavendish cultivar Grand Nain (GN). After screening primary transformants by PCR, five *RGA2* lines (RGA2-2, 3, 4, 5, and 7) and five *Ced9* lines (Ced9-17, 19, 21, 22, and 26), together with untransformed controls, were selected for Southern analysis. Each of the *RGA2* lines contained multiple transgene copies in addition to three endogenous *RGA2* homologs present in untransformed control plants (Fig. 1c). The five *Ced9* lines contained between one and many transgene copies with no endogenous homologs identified (Fig. 1d).

### Field assessment of transgenic banana plants

To determine whether these transgenes could confer TR4 resistance, we assessed the ten independent transgenic lines (RGA2-2, 3, 4, 5, and 7; Ced9-17, 19, 21, 22, and 26), along with five additional PCR-positive transgenic lines but for which Southern analysis had not been done (RGA2-6; Ced9-10, 15, 23, and 31), for resistance in a field trial over a 3-year period. The trial site was a commercial banana plantation in the Northern Territory of Australia where TR4 has become endemic and where Cavendish banana plants had previously been devastated by the disease. Non-transgenic controls included in the trial were the TR4-susceptible Cavendish cultivars GN and Williams, in addition to cultivars GCTCV 218 and DPM25 (Dwarf Parfitt Mutant). GCTCV 218 is a variant of Giant Cavendish selected in Taiwan for its tolerance to TR4^6^, whereas DPM25 is a ɣ-irradiated selection of Cavendish cultivar Dwarf Parfitt, which has resistance to another race of Foc, subtropical race 4 (STR4)^12^. The trial location has a tropical climate with about 90% of its annual rain usually falling during the wet season (November–April). The trial was planted during the wet season in early 2012, according to a randomized design, and run for 3 years, as banana is a perennial crop. To increase the inoculum pressure, infected plant material was buried between each plant.

**Fig. 2:**
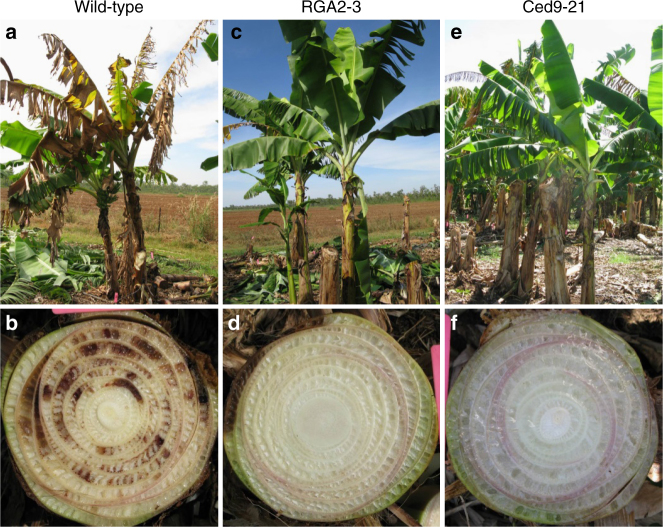
Characteristic symptoms of Foc TR4 in susceptible and resistant banana. External symptoms and reddish-brown internal vascular discoloration of Foc TR4 in infected WT Cavendish **a** and **b** compared with resistant transgenic lines RGA2-3 **c** and **d**, and Ced9-21 **e** and **f**

**Fig. 3:**
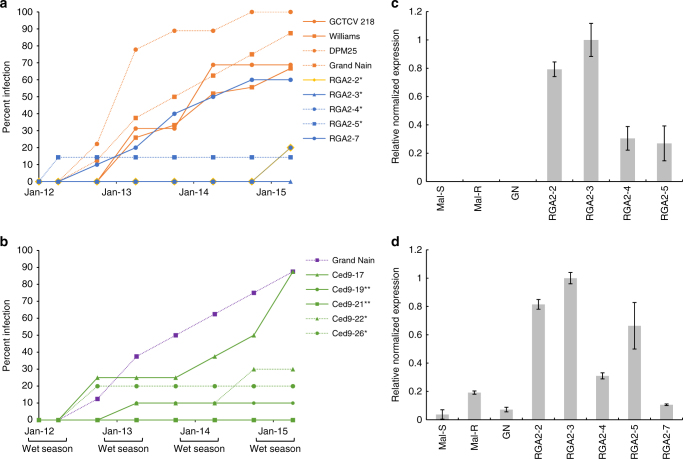
Disease incidence and gene expression analysis. **a, b** Levels of Foc TR4 infection in selected transgenic and WT banana plants throughout the 3-year field trial. **a** WT and RGA2 lines, and **b** WT and Ced9 lines. Wet seasons (November–April) are indicated. The number of biological replicates (*n*) of each independent line at the start of the trial is provided in Supplementary Table 1. Data points are percentage of biological replicates infected at time of assessment. *0.01 < *p* < 0.05, **0.001 < *p* < 0.01 at trial end (Tukey's HSD test). **c, d** Analysis of *RGA2* mRNA expression levels in transgenic and WT banana plants. **c** Analysis of transgene (*RGA2*-Nos) expression levels using primers designed to amplify a 96 bp fragment spanning the *RGA2* transgene/Nos terminator junction. **d** Analysis of *RGA2* transgene and endogenous mRNA expression levels using primers designed to amplify a 92 bp fragment of both the *RGA2* transgene and *RGA2* endogenous sequences. All values are normalized expression levels expressed relative to line RGA2-3. WT GN; TR4-susceptible *M. acuminata* ssp. *malaccensis* (Mal-S) and TR4-resistant *M. acuminata* ssp. *malaccensis* (Mal-R). A single biological replicate was analyzed with three technical replicates (*n* = 3). Data are mean ± SEM

The trial was regularly inspected for plants showing typical TR4 symptoms such as wilting and/or leaf yellowing (Fig. 2a). The pseudostems of symptomatic individuals were further examined for the presence of the reddish-brown vascular discoloration characteristic of TR4 infection (Fig. 2b). The disease status of the plants, based on this visual assessment, was recorded at regular intervals in both the wet and dry (May–October) seasons (Figs. 3a,b and Table 1). For the final assessment, the pseudostems from all of the surviving plants were scored for vascular discoloration and a selection of samples taken and tested for TR4 by a combination of fungal isolation and PCR-based assays^13,14^. Of the 118 samples, 107 were from plants showing no vascular discoloration and all tested negative for TR4. The 11 remaining samples came from plants with vascular discoloration and, of these, 10 tested positive for Foc TR4. This confirmed that vascular discoloration in the trial was more than 99% accurate as a diagnostic marker for infection by TR4.

**Table 1:** Accumulated rates of Foc TR4 disease throughout the 3-year field trial

Line	Number of plants in the field (*n*)	Chronological incidence of TR4 infection (percent of symptomatic plants)							
		January 2012	April 2012	October 2012	April 2013	October 2013	April 2014	October 2014	April 2015
GCTCV 218	16	0	0	0	31.3	31.3	68.8	68.8	68.8
Williams	27	0	0	0	25.9	33.3	51.9	55.6	66.7
DPM25	9	0	0	22.2	77.8	88.9	88.9	100	100
GN	8	0	0	12.5	37.5	50	62.5	75	87.5
RGA2-2	10	0	0	0	0	0	0	0	20*
RGA2-3	8	0	0	0	0	0	0	0	0*
RGA2-4	10	0	0	0	0	0	0	0	20*
RGA2-5	7	0	14.3	14.3	14.3	14.3	14.3	14.3	14.3*
RGA2-6	6	0	0	16.7	50	50	50	50	66.7
RGA2-7	10	0	0	10	20	40	50	60	60
Ced9-10	8	0	0	12.5	25	37.5	37.5	37.5	37.5
Ced9-15	10	0	0	0	10	10	20	20	50
Ced9-17	8	0	0	25	25	25	37.5	50	87.5
Ced9-19	10	0	0	0	10	10	10	10	10**
Ced9-21	9	0	0	0	0	0	0	0	0**
Ced9-22	10	0	0	0	10	10	10	30	30*
Ced9-23	10	0	0	0	10	10	10	10	20
Ced9-26	10	0	0	20	20	20	20	20	20*
Ced9-31	10	0	0	10	10	10	20	20	20

DPM25, Dwarf Parfitt Mutant; GCTCV, Giant Cavendish Tissue Culture Variants; GN, Grand Nain; HSD, honest significant difference; TR4, tropical race 4. Data are percentage of biological replicates infected at time of assessment. *0.01 < *p* < 0.05, **0.001 < *p* < 0.01 at trial end (Tukey's HSD test).

By the end of the trial, between 67 and 100% of all control plants were either dead or infected and all had displayed vascular discoloration. In general, disease developed earlier in the non-transgenic control plants, increased by about 20% per year, and was considerably faster during the wet seasons (Figs. 3a,b and Table 1). DPM25, which is noted for its resistance to Foc STR4^12^, appeared to be the most susceptible cultivar with all of its replicates becoming infected within 2.5 years, whereas the reportedly TR4-tolerant GCTCV 218^6^ was as susceptible as the Cavendish cultivar Williams. However, there was no statistical difference in the total proportion of symptomatic infected plants between any of the controls compared with the reference control, GN (Tukey's honest significant difference (HSD) test, *p* > 0.05). In contrast, 30% or fewer of plants of four of the five characterized RGA2 lines and four of the five characterized Ced9 lines were symptomatic during the 3-year trial. Symptom development in the four RGA2 lines, RGA2-2, RGA2-3, RGA2-4, and RGA2-5, was significantly lower than that in GN (Tukey's HSD test, 0.01 < *p* < 0.05), whereas RGA2-7 (60%) was not. Similarly, symptom development was significantly lower in the four Ced9 lines, Ced9-22, and Ced9-26 (Tukey's HSD test, 0.01 < *p* < 0.05), and Ced9-19 and Ced9-21 (Tukey's HSD test, 0.001 < *p* < 0.01) than GN, whereas Ced9-17 (87.5%) was not. Lines RGA2-3 and Ced9-21 appeared to be immune to TR4, as no internal (vascular discolouration) or external symptoms of the disease were observed in any plant throughout the 3 years (Figs. 2c–f, Figs. 3a, b, and Supplementary Table 1).

### Analysis of gene expression levels

To investigate the basis of the resistance, and because TR4 is a soil-borne pathogen, roots from the Ced9 and RGA2 lines were assayed for transgene expression. Quantitative analysis was only done on RGA2 lines, because, as a banana-derived gene, *RGA2*was considered to be the most suitable transgene for deregulation. Reverse transcriptase PCR (RT-PCR) showed that all five Ced9 lines expressed the transgene (Supplementary Fig. 1). For the RGA2 lines, quantitative RT-PCR (qRT-PCR), initially with primers that amplified the *RGA2* transgene only, was used to assess expression levels and a strong correlation was observed between *RGA2* expression level and the degree of TR4 protection (Fig. 3c). Subsequently, qRT-PCR was repeated but with primers that would amplify both the *RGA2* transgene and endogenous *RGA2* in GN (Fig. 3d). Again, the most resistant transgenic line (RGA2-3) was the highest expressor of RGA2 mRNA, whereas the other three TR4-resistant lines, RGA2-2 (20% infection), RGA2-4 (20% infection), and RGA2-5 (14.3% infection), also showed moderate to high levels of RGA2 expression. The most susceptible line RGA2-7 (60% infection) had the lowest expression levels, which were about tenfold less than RGA2-3. The level of expression in the highly susceptible Cavendish cultivar GN (87.5% infection) was also about tenfold lower than line RGA2-3. The observed inverse correlation between the proportion of infections and the expression level of *RGA2* in non-transgenic GN and the RGA2 transgenic lines was statistically significant (Pearson's correlation = −0.86, *t *= −3.37, df = 4, *p* = 0.028; Spearman’s rank correlation = −0.90, *S* = 66.45, *p* = 0.015). This inverse relationship persisted when only the RGA2 transgenic lines were considered but the correlation was no longer statistically significant (Pearson's correlation = −0.85, *t* = −2.811, df = 3, *p* = 0.067; Spearman’s rank correlation = −0.82, *S* = 36.42, *p* = 0.089).

Interestingly, RGA2 expression in TR4-resistant wild diploid *M. acuminata* ssp. *malaccensis* was more than fivefold greater than in its susceptible sibling but more than fivefold less than RGA2-3. Fortunately, enhancing levels of RGA2 or Ced9 expression does not appear to have a detrimental impact on bunch size in the transgenic lines. A visual rating of the size of available mature bunches (Supplementary Table 2) based on the number of fruit hands per bunch showed no statistical difference between healthy control and transgenic GN (*χ* ^2^ = 31.7, df = 28, *p* = 0.29).

### Possible resistance mechanisms and future research

**Fig. 4:**
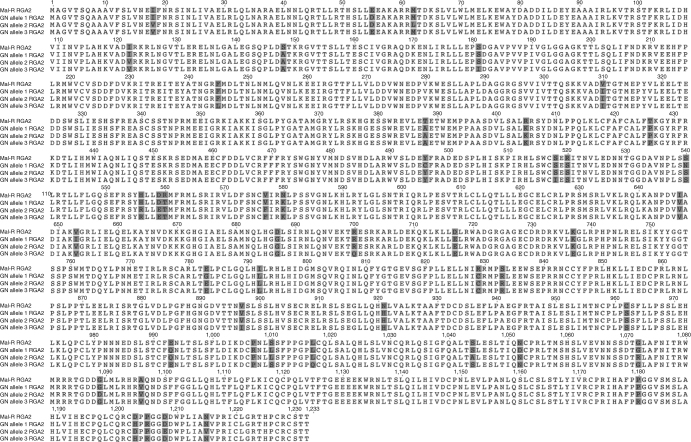
Protein sequence alignment of the *RGA2* transgene sequence from TR4-resistant *M. acuminata* ssp. *malaccensis* (Mal-R) and the three consensus *RGA2* homologous sequences from WT GN. Amino acid differences are highlighted

These results indicate that the Cavendish cultivar GN possesses endogenous *RGA2* loci, which are not sufficiently expressed to confer TR4 protection. Given the close relationship of GN and the other Cavendish cultivars Williams, GCTCV 218, and DPM25, it is probable that the latter clones also possess *RGA2* loci. Increasing RGA2 levels by transgenesis may reduce TR4 infection, possibly through an R-gene-like cascade pathway. The gene(s) responsible for the Foc TR4 resistance in *M. acuminata* ssp. *malaccensis* have not been identified but *RGA2* is a possible candidate. It seems highly unlikely that the observed resistance to TR4 in our *RGA2* lines is due to somaclonal variation, because somaclonal variants that exhibit even tolerance to TR4 are rare, yet we identified four out of five independent *RGA2* lines with resistance. Southern blot analyses indicated that even the highly susceptible GN contains three *RGA2*-like sequences (Fig. 1c). Cloning and analysis of these *RGA2*-like sequences confirmed the presence of three *RGA2* homologs, which were between 98.6 and 98.7% homologous with *RGA2* at the nucleotide level resulting in between 28 to 32 amino acid changes (Fig. 4). Further analysis indicated that these were three alleles of a single copy *RGA2* homolog. To determine whether low expression levels of the homologs, amino acid differences, or a combination of the two renders the three *RGA2* homologs ineffective, GN has been transformed with each homolog, under the control of the Nos promoter, and the response of the transgenic lines to TR4 will be assessed in future field trials. The mode of action of *Ced9*-mediated TR4 resistance in transgenic bananas is also unclear. Functioning as an anti-apoptosis gene, it may be preventing fungal-induced cell death and contributing to the maintenance of organelle homeostasis.

Previously, transgenic resistance to Foc race 1 in banana has been reported from glasshouse testing using either anti-apoptosis genes^8,15^ or RNA interference targeting essential Foc genes^16^. This is the first report of Fusarium wilt resistance in transgenic bananas in the field. It is a very significant step toward averting the collapse of Cavendish-based banana export production and at the same time protecting a major subsistence crop. We are about to commence another more expansive field trial containing our RGA2 lines, as well as many more new Cavendish (cultivars Williams and GN) lines transformed with *RGA2*, to identify lines that are suitable for progression through to commercial release. Further, the discovery that Cavendish encodes three *RGA2* homologs provides the exciting prospect of using gene editing technology to generate TR4 resistance by enhancing the expression of these endogenous genes with or without also editing the coding sequence.

## Methods

### Plant transformation and characterization

Binary vectors containing either the *C. elegans* gene *Ced9* under the control of a maize Ubi-P and a cauliflower mosaic virus 35s terminator (35S-T)^8^ or the *M. acuminata* ssp. *malaccensis* *RGA2* under the control of the *Agrobacterium tumefaciens* Nos-P and terminator sequences^17^ were used in this study *M. acuminata* Cavendish cv. GN (AAA subgroup) embryogenic cell suspensions were prepared from immature male flowers and transformed using the centrifugation assisted *A. tumefaciens*-mediated method^18^. Following selection, plants derived from single embryos were regenerated and were screened for the presence of the respective transgene by PCR using specific primers. A total of six *RGA2* and nine *Ced9* lines were generated, and up to 10 replicates of each transgenic line was multiplied in tissue culture for field analysis. Both the number of transgenic lines and the number of replicates per line permitted in the field trial was limited by the licence conditions (DIR 107) imposed by the Office of the Gene Technology Regulator (OGTR). Plants derived from non-transformed cell suspensions were also generated as controls. Before field planting, tissue-culture plants were acclimatized in a secure shade house over a 3-month period by which time they had reached a height of ~35 cm.

### Field trial design

The field trial was conducted on a commercial banana plantation site located at Lambells Lagoon, Northern Territory, Australia. The site had previously been used to grow Cavendish banana plants and has a history of high incidence of TR4 infection. The field trial comprised two plantings, the first in January 2012 and the second in May 2012. Owing to the seriousness of the threat of TR4 to the Australian banana industry, it took 8 years from the initial generation of the transgenic lines to identify a suitable field trial location and obtain permission from the plantation owner, the biosecurity regulators in both Queensland and the Northern Territory, and the OGTR to conduct this trial. Unfortunately, the trial was only conducted for 3 years, not the intended 5 years, because of a forced quarantine termination order due to another disease.

Transgenic lines were planted in a randomized design, with rows containing blocks of ten transgenic plants and each block separated by four non-transgenic control plants. The control plants included cell line control plants (cv Cavendish selections GN and/or Williams) from QUT in addition to tissue culture plants of Williams, GCTCV 218 and DPM25 supplied by Mission Beach Tissue Culture Nursery, Queensland. To increase the level and uniformity of inoculum pressure, a small segment of pseudostem taken from TR4-infected banana plants growing outside the trial site was buried between each plant.

When a banana is planted, the plant crop, a pseudostem comprising the petioles of leaves grows from the basal corm with the vegetative meristem remaining at the base of the pseudostem. When flowering is initiated, the meristem is pushed up through the center of the pseudostem, the fruit bunch emerges through the top of the pseudostem and develops through to maturity. After the bunch is harvested, this initial pseudostem dies back and another new pseudostem, known as the first ratoon, grows from a different meristem on the corm. This process can be repeated indefinitely. The trial consisted of the plant crop and up to three ratoon crops.

### Assessment of symptoms and bunch size

Plants were regularly assessed over three crop cycles (~3 years) for the development of characteristic external symptoms of Fusarium wilt disease^1^. The pseudostem of plants exhibiting typical external symptoms was inspected to confirm the presence of characteristic internal reddish-brown vascular discoloration associated with infection by Foc. If a plant shows the characteristic external symptoms of wilting and/or yellowing of their leaves, it almost invariably develops severe vascular necrosis followed by death of that pseudostem. In some instances, the symptomatic pseudostem would die but an apparently healthy ratoon pseudostem would grow from the basal corm. The first observation of symptoms was recorded for each plant; however, ratoons from diseased plants were allowed to develop naturally until the completion of the trial period. At the completion of the trial period, all plants were inspected for external and internal symptoms. The presence of the internal vascular discoloration, which is highly characteristic of Foc TR4 infection, was assessed in all remaining plants by cutting the pseudostem of all plants ~0.5 metres above ground level. Where vascular discoloration was observed, a pseudostem sample of ~3 cm × 1 cm was taken from the leading edge of the discoloration. Where no vascular discoloration was observed, a similar sample was taken from an equivalent area. Pseudostem samples from 118 plants were collected and were sent by air freight to Wageningen UR, Plant Sciences Group, The Netherlands, for confirmatory diagnosis of Foc infection.

To avoid any bias, all plants in the trial were assigned a unique Field Trial Number which contained no identifying information. Further, the assessment of disease symptoms during the 3 year trial period was done independently by the farm manager (MS) who has more than 20 years experience managing a TR4-infested commercial banana farm. At the completion of the trial, assessment for internal and external symptoms was done by two of the authors (JD and RH).

At various times during the trial, the size of mature fruit bunches on healthy transgenic and non-transgenic plants was visually assessed and rated into three categories, <6, 6–8, or >8 hands per bunch.

### Diagnosis of Foc infection

The pseudostem samples were used for a combination of fungal isolation and molecular diagnostic analyses by PCR^13,14^ and quantitative PCR (qPCR kit: “Foc TR4 DNA identification by Real-Time PCR, Clear Detections”, The Netherlands). For DNA isolation from plant samples, small pieces of pseudostem tissue were selected and subsequently lyophilized in an Epsilon 1-4/2–4 LSC plus freeze dryer (Martin Christ) for 3 days. For a subset of 49 samples that were collected from the above mentioned materials, 2–4 pieces of the same tissue were sterilized with 1% hypochlorite, rinsed with Milli-Q water, dried in filter paper, and placed on Komada medium for fungal re-isolation^13^. After 5 days, emerging Foc colonies were sampled for PCR analysis^13^, as well as qPCR. Total plant and fungal DNA was extracted using the Sbeadex maxi plant Kit (LGC Genomics). DNA extractions from all plant and fungal samples were independently repeated at least twice. Analytical PCRs were also technically repeated twice. qPCR was conducted in a total volume of 20 µL reaction mixture containing 10 µL of Clear Detections PCR mix, 2 µL of Foc TR4 primer set, 3 µL of PCR enhancer, 0.2 µL of ROX reference dye II (Takara), 3 µL of DNA template, and Milli-Q water in a 7500 Real-Time PCR system (Applied Biosystems). Thermal cycling conditions for amplification were an initial enzyme activation at 95 °C for 3 min, followed by 35 cycles each consisting of 95 °C for 10 s, 63 °C for 60 s, and 72 °C for 30 s. Finally, for the PCR melt curve 0.2–0.5 °C, steps at 72–95 °C were included. The amplification results were analyzed with 7500 Real-Time PCR software v 2.3 (Applied Biosystems).

### Transgene expression analysis

Owing to quarantine restrictions, we were unable to transport samples from banana plants within Australia for analysis. Therefore, transcript analyzes were done using samples taken from the original mother plants stored in tissue culture at QUT, Brisbane, Australia. Tissue culture plants of wild-type (WT) TR4-susceptible and resistant *M. acuminata* ssp. *malaccensis* were used as controls^19,20^.

Total RNA was extracted from 200 mg of root tissue using a protocol^21^ that was modified by increasing the tissue:extraction buffer ratio to 8, including a centrifugation step (18,000x*g* for 5 min) before the initial solvent extraction and the omission of phenol from all extraction steps. RNA (3 μg) was DNase-treated using an RQ1 RNase-free DNase Kit (Promega) and DNA-free RNA samples (1.8 μg) were reverse-transcribed to complementary DNA using an oligo(dT)18 primer and the GoScript Reverse Transcription System (Promega) according to the manufacturer’s instructions. Subsequently, cDNA samples were diluted either 1:10 (v/v) or 1:8 (v/v) in RNase-free water before use in RT-PCR and qRT-PCR, respectively. To ensure complete removal of potential gDNA contamination in our samples before RT-PCR and qPCR, total RNA, DNase-treated RNA, as well as cDNA was tested by PCR using the cyclophilin (*CYP*) housekeeping gene primers (Supplementary Table 3).

Reaction mixes for RT-PCR contained 1 × GoTaq Green master mix (Promega), 0.25 µM of each primer (Supplementary Table 3), diluted cDNA (2 µL), and nuclease-free water in a final volume of 20 µL. Thermal cycling conditions included a 2 min denaturation step at 94 °C followed by 35 cycles of 94 °C for 20 s, 55–62 °C (primer dependent) for 30 s, and 72 °C for 1 min Kbp^-1^ of expected amplicon size, and a final extension at 72 °C for 5 min.

qRT-PCR was performed on a CFX384 Touch Real-Time PCR Detection System (Bio-Rad) using the SYBR Green I technology. Per 10 μL reactions, 2.5 μL of diluted cDNA was added to 1 × GoTaq qPCR Master Mix (Promega) premixed with primers (Supplementary Table 3) at a final concentration of 0.2 μM. The following amplification program was used: Hot-Start polymerase activation at 95 °C for 2 min, followed by 45 cycles of 10 s denaturation at 95 °C and 30 s annealing/extension at 60 °C. At the end of each run, a dissociation curve was produced from 65–95 °C, to confirm the specificity of the amplicon from each primer set. A standard curve of eight serial two-fold dilutions of cDNA was used to determine the qPCR efficiency of each of the primer sets used^22^. All PCR reactions displayed a correlation coefficient *R* ^2^>0.98 and efficiencies >99% (Supplementary Fig. 2). All samples were analysed in triplicate and each run included triplicate non-template control reactions for each of the primer sets used on that run. In addition, selected samples from each run were electrophoresed through 2 % agarose gels to validate production of a single amplicon.

Relative expression levels were calculated using CFX Manager 3.1 (Bio-Rad) software and the ΔΔCT method^23^. Ct data obtained from target gene of interest were normalized using Ct values from the two stable reference genes *CYP* and ribosomal protein *S2* (*RPS2*), and expressed relative to the values of line RGA2-3. All primers were designed using the Primer3Plus freeware (http://www.bioinformatics.nl/cgi-bin/primer3plus/primer3plus.cgi).

### Southern blot analysis

For determination of transgene copy number integration by Southern analysis, total nucleic acid was extracted^24^ from banana leaf tissue, treated with RNAse A, and 15 μg aliquots of genomic DNA were digested overnight with 20 U of restriction enzyme *Hin*dIII or *Xma*I (New England Biolabs) overnight at 37 °C. Digested DNA was electrophoresed through 0.9% agarose gels, transferred to a nylon membrane (Roche), and UV cross-linked. Gene-specific probes were amplified by PCR using Taq DNA polymerase (Sigma-Aldrich) in reaction mixes containing the appropriate primers (Supplementary Table 3), 2 ng of plasmid template and DIG PCR labeling mix (Roche). Hybridization of the probe was done under standard conditions^25^ and detection was achieved using CDP-star (Roche), as per the manufacturer’s instructions. X-ray films (Fuji) were exposed for up to 1 h depending on signal intensity and developed manually.

### Isolation and analysis of endogenous Cavendish RGA2 homologs

The entire open reading frame of the endogenous Cavendish *RGA2* sequence was amplified from total nucleic acid prepared^24^ from leaf tissue collected from WT GN plants. Primers RGA2geneF (5′-ATGGCTGGTGTCACATCACAGGCAG-3′) and RGA2geneR (5′-TCAGGTGGTGCTACAGCGACATGG-3′) were designed based on the *M. acuminata* ssp. *malaccensis RGA2* sequence^9^. PCR was carried out using either GoTaq Long Master Mix (Promega) or Expand Hi-Fidelity Enzyme Mix (Roche). GoTaq Long PCR mixtures contained 20 μL 2× GoTaq Long Master Mix, 10 ρmol of each primer, 0.5 μL TNA extract, and 17.5 μL water. Cycling conditions were 95 °C for 2 min, followed by 35 cycles of 95 °C for 15 s, 50 °C for 15 s, 65 °C for 8 min, and a final extension at 72 °C for 10 min. PCR using Expand was carried out according to the manufacturer’s instructions, with cycling as above except for extension carried out at 68 °C. PCR products were analyzed on 1% agarose gels and visualized using SYBR safe DNA gel stain (Thermo Fisher Scientific). PCR products of the expected size were excised from gels, ligated into pGemT Easy (Promega), and heat-shock transformed into competent *Escherichia coli* XL-1 Blue cells (Invitrogen). Blue/white selection was used to identify putative recombinant colonies, with six clones derived from each PCR amplification selected and grown in overnight cultures. Plasmid DNA was purified using the Wizard *Plus* SV Miniprep DNA purification system (Promega) and digested using *Not*I to identify clones with inserts of the expected size. Plasmids were then sequenced using the BigDye Terminator mix v3.1 (Applied Biosystems) according to the manufacturer’s instructions, with sequence reads generated at the Queensland University of Technology Molecular Genetics Research Facility. Specific primers were used to completely sequence the inserts in both directions. Raw sequence reads were compiled into full-length sequences using the VectorNTI Advance V11 software (Life Technologies) program ContigExpress, whereas sequence alignments were carried out using AlignX. Consensus sequences of the WT GN *RGA2* homologs were compared with the *M. acuminata* spp. *malaccensis* *RGA2* sequence, to determine the level of nucleotide and amino acid sequence similarity.

### Statistical analysis

Differences in total proportions of infected plants between the treatment and control groups, and between lines, were assessed using single and mixed effects generalized linear models and corresponding analyses of variance, assuming a binomial response and with the respective addition or subtraction of 0.5 for 0 or total infections.

Pairwise comparisons between treatments and lines, adjusted for multiple comparisons, were evaluated using Tukey's HSD test. Model fit was assessed using a *χ* ^2^-test of deviance and the Akaike information criterion (AIC).

The equivalence of ratings of fruit bunches from control, RGA2 lines and the Ced9 lines as number of hands in the bunch (<6, 6–8, and >8) was assessed using a *χ* ^2^-test of homogeneity.

The hypothesis of a (linear) relationship between the proportion of infections and the expression level for each of the RGA lines and the GN control line was assessed using parametric (Pearson) and nonparametric (Spearman) correlations, and the associated tests.

Statistical significance was asserted at the 5% level (*p* < 0.05). The analyses were undertaken in R using base statistical functions and the packages lme4, lmer, and multcomp.

### Data availability

The authors declare that all data supporting the findings of this study are available within the article and/or its Supplementary Information file. All relevant data are also available from the authors upon request.

## Electronic supplementary material

Supplementary Information
