# Supplementary material for: Transgenic Cavendish bananas with resistance to Fusarium wilt tropical race 4
Source: Nat Commun. 2017 Nov 14;8:1496. doi: 10.1038/s41467-017-01670-6 (PMC5684404; doi:10.1038/s41467-017-01670-6)
Supplement: Supplementary file 1 — Supplementary Information [file 41467_2017_1670_MOESM1_ESM.pdf]

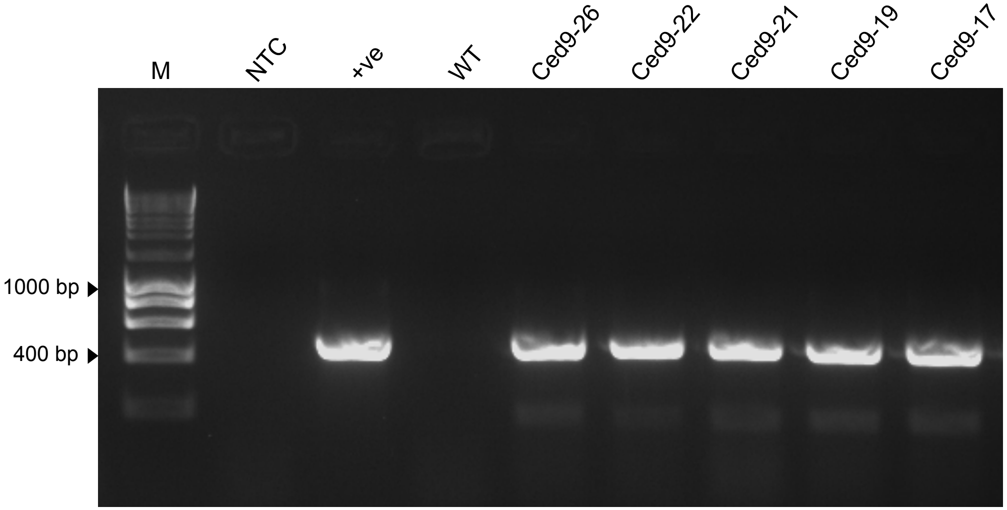

Supplementary Figure 1. Reverse transcriptase-PCR analysis of *Ced9* mRNA expression in selected lines. No-template control (NTC); plasmid DNA positive control (+ve); wild-type (WT) and DNA molecular weight marker HyperLadder I (Bioline) (M).

a

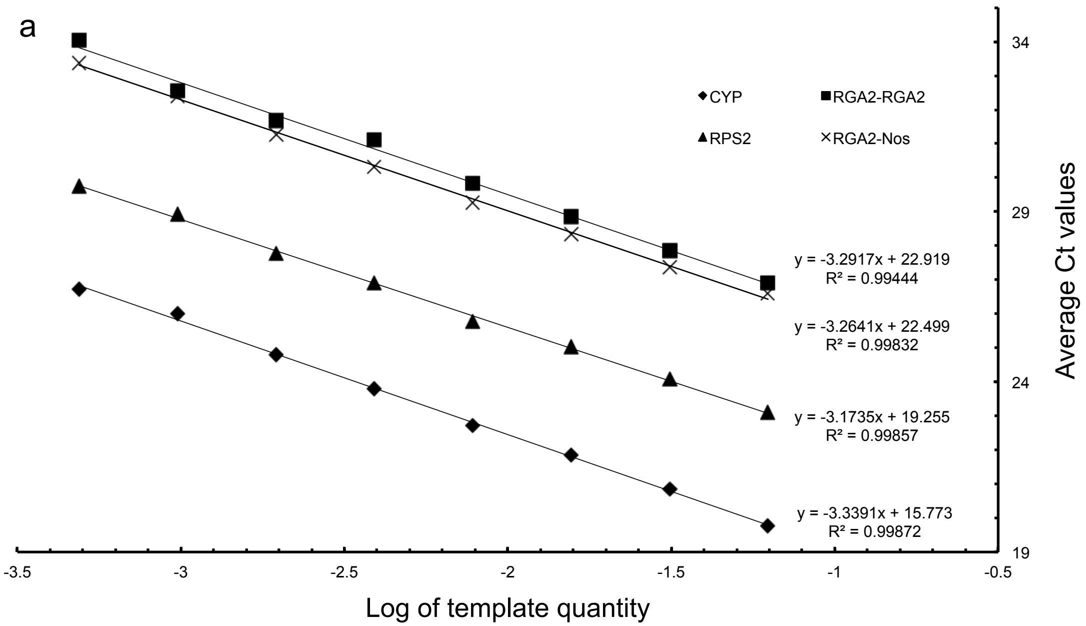

b

Target gene	Efficiency calculation	Efficiency (%)
RGA2	1+10^(-1/-3.2917)	101.3
RGA2-Nos	1+10^(-1/-3.2641)	102.5
CYP	1+10^(-1/-3.3391)	99.3
RPS2	1+10^(-1/-3.1735)	106.6

Supplementary Figure 2. Efficiency of primer pairs used for quantitative reverse transcriptase-PCR.
a. Dose response curve of primer pairs used to amplify each target gene.
b. Calculation of PCR efficiency for each primer pair.

Supplementary Table 1. Status of individual replicates of the transgenic lines

Transgenic line	Biological replicate	Date replicate was generated	Date field trial planted	Date field trial ended	Disease status in field trial at April 2015 (trial end)	Percent infection in field trial at April 2015 (trial end)	Date expression level measured
**RGA2-2**	1	Apr-04	Jan-12	Apr-15	Healthy	20%	May-16
	2				Healthy		
	3				TR4 infected		
	4				Healthy		
	5		May-12		TR4 infected		
	6				Healthy		
	7				Healthy		
	8				Healthy		
	9				Healthy		
	10				Healthy		
**RGA2-3**	1	Apr-04	Jan-12	Apr-15	Healthy	0%	May-16
	2				Healthy		
	3				Healthy		
	4				Healthy		
	5				Healthy		
	6		May-12		Healthy		
	7				Healthy		
	8				Healthy		
**RGA2-4**	1	Apr-04	Jan-12	Apr-15	Healthy	20%	May-16
	2				Healthy		
	3				Healthy		
	4				Healthy		
	5		May-12		TR4 infected		
	6				TR4 infected		
	7				Healthy		
	8				Healthy		
	9				Healthy		
	10				Healthy		
**RGA2-5**	1	Apr-04	Jan-12	Apr-15	Healthy	14.3%	May-16
	2				Healthy		
	3				Healthy		
	4				Healthy		
	5				Healthy		
	6				Healthy		
	7				TR4 infected		
**RGA2-6**	1	Apr-04	Jan-12	Apr-15	TR4 infected	66.7%	Not done
	2				TR4 infected		
	3		May-12		Healthy		
	4				TR4 infected		
	5				Healthy		
	6				TR4 infected		
**RGA2-7**	1	Apr-04	Jan-12	Apr-15	TR4 infected	60%	May-16
	2				TR4 infected		
	3				TR4 infected		
	4				Healthy		
	5				TR4 infected		
	6				TR4 infected		
	7				Healthy		
	8		May-12		Healthy		
	9				TR4 infected		
	10				Healthy		

Supplementary Table 1. Continued

Transgenic line	Biological replicate	Date replicate was generated	Date field trial planted	Date field trial ended	Disease status in field trial at April 2015 (trial end)	Percent infection in field trial at April 2015 (trial end)	Date expression level measured
**Ced9-10**	1	Sep-04	Jan-12	Apr-15	TR4 infected	37.50%	Not done
	2				Healthy		
	3				TR4 infected		
	4				Healthy		
	5				TR4 infected		
	6				Healthy		
	7				Healthy		
	8		May-12		Healthy		
**Ced9-15**	1	Sep-04	Jan-12	Apr-15	Healthy	50%	Not done
	2				TR4 infected		
	3				TR4 infected		
	4				TR4 infected		
	5				Healthy		
	6		May-12		TR4 infected		
	7				Healthy		
	8				Healthy		
	9				TR4 infected		
	10				Healthy		
**Ced9-17**	1	Sep-04	Jan-12	Apr-15	TR4 infected	87.5%	Not done
	2				TR4 infected		
	3				TR4 infected		
	4		May-12		Healthy		
	5				TR4 infected		
	6				TR4 infected		
	7				TR4 infected		
	8				TR4 infected		
**Ced9-19**	1	Sep-04	Jan-12	Apr-15	Healthy	10%	Not done
	2		May-12		TR4 infected		
	3				Healthy		
	4				Healthy		
	5				Healthy		
	6				Healthy		
	7				Healthy		
	8				Healthy		
	9				Healthy		
	10				Healthy		
**Ced9-21**	1	Sep-04	Jan-12	Apr-15	Healthy	0%	Not done
	2				Healthy		
	3				Healthy		
	4				Healthy		
	5		May-12		Healthy		
	6				Healthy		
	7				Healthy		
	8				Healthy		
	9				Healthy		

Supplementary Table 1. Continued

Transgenic line	Biological replicate	Date replicate was generated	Date field trial planted	Date field trial ended	Disease status in field trial at April 2015 (trial end)	Percent infection in field trial at April 2015 (trial end)	Date expression level measured
**Ced9-22**	1	Sep-04	Jan-12	Apr-15	Healthy	30%	Not done
	2				TR4 infected		
	3				Healthy		
	4				Healthy		
	5				Healthy		
	6				Healthy		
	7				TR4 infected		
	8		May-12		TR4 infected		
	9				Healthy		
	10				Healthy		
**Ced9-23**	1	Sep-04	Jan-12	Apr-15	Healthy	20%	Not done
	2		May-12		Healthy		
	3				Healthy		
	4				TR4 infected		
	5				Healthy		
	6				Healthy		
	7				Healthy		
	8				Healthy		
	9				Healthy		
	10				TR4 infected		
**Ced9-26**	1	Sep-04	Jan-12	Apr-15	Healthy	20%	Not done
	2				Healthy		
	3				Healthy		
	4				Healthy		
	5				Healthy		
	6				TR4 infected		
	7				TR4 infected		
	8		May-12		Healthy		
	9				Healthy		
	10				Healthy		
**Ced9-31**	1	Sep-04	Jan-12	Apr-15	Healthy	20%	Not done
	2				TR4 infected		
	3		May-12		Healthy		
	4				Healthy		
	5				Healthy		
	6				Healthy		
	7				Healthy		
	8				Healthy		
	9				TR4 infected		
	10				Healthy		

Supplementary Table 2. Assessment of bunch size of individual replicates within transgenic lines and Grand Nain controls

Line	Number of hands per bunch		
	< 6	6-8	> 8
Grand Nain (n=6)		5	1
RGA2-2 (n=3)		3	
RGA2-3 (n=5)	1	2	2
RGA2-4 (n=4)		4	
RGA2-5 (n=6)		4	2
RGA2-6 (n=2)		1	1
RGA2-7 (n=5)		2	3
Ced9-10 (n=6)		6	
Ced9-15 (n=5)		2	3
Ced9-17 (n=3)		3	
Ced9-19 (n=2)		2	
Ced9-21 (n=2)	1	2	
Ced9-22 (n=4)	1	3	1
Ced9-23 (n=2)		2	
Ced9-26 (n=6)		3	3
Ced9-31 (n=2)		1	1

Supplementary Table 3. Sequences of primers used in this study

Target sequence	Primers	Sequence (5' → 3')	Purpose	Amplicon length (bp)
CYP	qCYP-F1	TGTCTTAGGAGGATGTAGAGGAGC	PCR, RT-PCR and qRT-PCR	182
	qCYP-R1	GGCTCCTGCTGACGATAATGAC		
RPS2	qRPS2-F	ACTCAACCGTCTTCCCAAAAG	qRT-PCR	110
	qRPS2-R	TCACAATATCAGGCAATCCCG		
RGA2	qRGA2nat-F	GAACAGGTGACGACGGCTTG	qRT-PCR	92
	qRGA2nat-R	AAACTGGAGGAAGGTGAGGTGT		
RGA2	RGA2gene seq2	ATGATGTGTGGAACGAAGACCC	PCR probe generation	486
	RGA2gene seq4	CCTTTCGAAACCTGTAGCCC		
RGA2-Nos	qRGA2trans-F	GTCGCTGTAGCACCACCT	qRT-PCR	96
	qNOS-R5	AAGACCGGCAACAGGATTC		
Ced9	Ced-9F2	GATGGCGACTGGCGAGATGAAG	RT-PCR	484
	Ced-9R2	GAAACCGCCGAACGAGATTAGACC		
Ced9	CED9 AsiSI fwd	GCGCGATCGCATGGCGACACGCTGCACG	PCR probe generation	862
	CED9 SbfI rev	GCCCTGCAGGTTACTTCAAGCTGAACATCATCC		
